# Lessons from the field: The role of agility in a coproduction project encompassing the COVID‐19 pandemic

**DOI:** 10.1111/hex.13372

**Published:** 2021-10-05

**Authors:** Rebecca Pritchard, Sonal Bhavsar, Pamela Campbell‐Morris, Prafulla Modi, Marie Nugent, Jason Hughes

**Affiliations:** ^1^ University of Edinburgh Edinburgh UK; ^2^ University Hospitals of Leicester NHS Trust Leicester UK; ^3^ NIHR Leicester BRC, Cardiovascular Research Centre Glenfield Hospital Leicester UK; ^4^ University of Leicester Leicester UK

**Keywords:** citizen science, community research, COVID‐19, priority‐setting exercise

## Abstract

**Aim:**

We reflect on our experiences of coproducing a redesigned, COVID‐safe priority‐setting activity at a time of shifting priorities and upheaval to gain insight into good practice.

**Method:**

The project team documented the experience of adapting to COVID‐19 through the reflective project evaluation. We reflect on how COVID disrupted coproduction through radically shifting personal and professional priorities and the implications for good practice.

**Results:**

Our experiences highlighted the role of agility, management capacity, social capital and power in coproduction.

**Conclusions:**

COVID‐19 disrupted and enabled coproduction, compounding tensions and serving as the basis to transcend them. The pandemic created new demands on institutions that initially prompted withdrawal to established power, and team members which redefined them in relation to each other. Shifting priorities and demands forced team members into new, and out of former, roles coming into conflict with enduring power dynamics articulating constructs of expertise and authority in the institutional structure. We consider how the tensions found expression: as governance and human resource concerns, problems with authorizing payments, challenges in institutionally accommodating community researchers and the exclusion of some from participation.

## 1 INTRODUCTION

Coproduction has some successes in engaging seldom‐heard communities and individuals,^1^ typically attributed to the intrinsic rewards and ability of the process to answer complex human motivations.^2^ As such, it has potential to enhance priority‐setting activities that establish the needs of a community of people, typically united by the experience of a health condition, using iterative focus groups to agree priorities by consensus (https://www.jla.nihr.ac.uk/about-the-james-lind-alliance/about-psps.htm, 26 February 2021).

In this piece of work, we reflect on adaptation of a priority‐setting activity. The project received funding in December 2019 to run a priority‐setting project exploring the health and research priorities of Leicester neighbourhoods. Priority data were to be collected by neighbourhood representatives themselves and jointly interpreted with institutional researchers. The pre‐COVID project sought to coproduce knowledge of local priorities, with institutional and community stakeholder.

The project plan was to recruit and develop community researchers to host focus groups at public engagement events delivered as part of a programme to engage seldom‐heard groups in health science using community arts methods successfully used in climate science engagement.^3^ They include dance classes and family activity days, as well as more conventional approaches, such as townhall meetings and advisory groups.

COVID‐19 forced significant project revision at a time of upheaval. Planned activities became unfeasible as lockdown began in April 2020. With key insights into the reality ‘on the ground’ within neighbourhoods, community researchers were pivotal in the redesign and led on participant‐facing redesign, with institutionally derived team members tackling the behind‐the‐scenes governance and bureaucracy. The community researchers designed socially distanced data collection. Institutional researchers negotiated processes for expression of interest, consent and documentation with the university.

The planned project was significantly disrupted and enabled by the pandemic. Agility and flexibility played a role in enabling and obstructing adaptation to challenges including a shift to remote working and associated challenges for governance arrangements, changes in workload and priority for all team members and an inevitable shift in the health concerns of communities.

As one of the most diverse (and deprived) cities in Europe, Leicester constitutes an ideal environment to explore the complex interdependencies between diversity, place, power, inequality, identity, issues of health and well‐being, concerns and outcomes. Both University Hospitals of Leicester NHS Trust and the University of Leicester seek to explore these interdependencies through a strategic shift towards community‐centric priority‐setting and practice that is coproduced to be locally responsive. Moreover, the university and hospital seek to develop sustained, collaborative, citizen‐centric engagement with local communities to coproduce policies and practices that effectively target the structural inequalities and disadvantages in health that underpinned the high rate of COVID‐19 infection in the city, which was, during the summer of 2020, 15× higher than the rate in England as a whole (https://www.leicester.gov.uk/your-council/coronavirus/coronavirus-in-leicester-latest-news/coronavirus-data-for-leicester/, 26 February 2021).

Research into factors underpinning the high rate of infection in Leicester is ongoing. The city's high prevalence of conditions that place one at risk (e.g., type 2 diabetes), communication challenges and diverse patterns of daily life including higher prevalence of multigenerational households, which occur in relation to the intersectionality of risk factors associated with exclusion and social deprivation, exacerbated COVID rates.^4^ Similarly, the role of high levels of low‐paid, insecure, key‐work employment in the health, social care, transport and manufacturing sectors has been acknowledged.^5^

The circumstances of the COVID‐19 pandemic presented a unique opportunity for coproduction. Against a backdrop of austerity, communities experienced fluctuations in social capital; people were furloughed, engaged in additional (child) care responsibilities and working from home. Communities spontaneously coproduced responses to the pandemic which maintained functionality, and tolerability.^6^

Though COVID‐19 represents a unique health crisis in the 21st century, it does share features of dominant concern in health sciences and healthcare—lifestyle diseases and climate change—as *wicked problems*. These are characterized by complexity, uncertainty, high impact on society, controversy, multiple stakeholders and the associated diverse values and goals;^6^ they articulate a postmodern science that dismantles disciplinary boundaries and the social constructs of knowledge and expertise.^7^ Coproduction has had some success in tackling wicked problems by engaging diverse, nontraditional expertise, creating better information, improving compliance and building trust in institutions and authorities.^8^ By contrast, typical institutional structures comprising ‘stovepipe systems’ that silo resources and expertise based on institutional characteristics (e.g., HR functions, finance functions) have posed obstacles to such coproduction.^9^ Reflections on coproduction throughout the pandemic may help inform future approaches to wicked issues.

Coproduction in commercial settings is ethically complex and potentially exploitative, with the primary (financial) benefit accrued to developers and investors by using an essentially unpaid workforce to drive creativity.^10^ Coproduction within the public sector is typically, though not exclusively,^11^ viewed as less asymmetric and therefore as a positive, democratically empowering, though still complex, activity in which exploitation is seen as more the result of bad practice than intrinsic.^12^, ^13^, ^14^ This assumes that, within the public sector, benefits of coproduction are accrued to citizens, with improved cost‐efficiency releasing funds to be used elsewhere in public services. As such, the National Institute for Health Research (NIHR) considers coproduction as a public involvement priority and a natural development of collaborative practices (https://www.nihr.ac.uk/documents/about-us/our-contribution-to-research/how-we-involve-patients-carers-and-the-public/Going-the-Extra-Mile.pdf, 26 February 2021).

Public service administration has developed from traditional, authoritarian administration through marketization into new public management and increased partnership working into new public governance. The impact and processes of coproduction and indeed the potential for and of coproduction have altered according to the context into the partnership model typically understood to represent good practice. However, shifts towards an altered model of public service administration described as communitarianism, which sees reduced investment in services leaving the community to meet needs with insufficient resources and support, have the potential to lead to damaging impacts of coproduction^2^ and leave communities feeling that the government has dumped sole responsibility^8^ and associated costs^15^ onto them. This potential for negative impact must be recognized and managed to avoid using a potentially powerful process to abdicate institutional and governmental responsibility.

It is therefore imperative to acknowledge the factors underpinning effective coproduction, which is a challenge as the existing literature comprises predominantly grey policy papers and is drawn from diverse socioeconomic and political cultures, and in respect of both commercial and public sector activity, making generalization difficult. Voorbergs’ review of predominantly grey policy papers identified compatibility of institutional systems with coproduction as a facilitator and institutional risk aversion as a barrier.^14^ Jaspers and Steen found coproduction to be facilitated by adaptive legislative frameworks, institutional resource allocation, alignment between institutional and community players and flexible governance in a project exploring the use of vacant spaces for short‐term coproduction projects in Flanders.^16^ Bovairds’ examination of case studies from Europe and the United States identified that failure to deliver these facilitators culminates in institutional resistance.^1^ Institutional structures are likely therefore to have the potential to facilitate or obstruct coproduction^17^ as a function of management capacity, which determines agility of systems in institutions.^18^

Within communities, and impacted by the political and institutional reality, social capital^6^, ^18^ in the form of an opportunity for learning and development, motivation as a function of appreciation and acknowledgement and ability to navigate ‘the system’^14^, ^16^ were found to positively impact effectiveness in diverse examples.

Though the NIHR encourages a pragmatic definition of coproduction, there is consensus that simply ‘really good collaboration’ is not coproduction, in contrast to the approach in the wider public service literature.^2^ Rather, coproduction in health sciences is seen as a deliberative process, founded on the principles of sharing power including awareness of power dynamics and their active management; including all perspectives and skills relevant to the project; respecting and valuing the knowledge of all those working together on the project; reciprocity by which all participants in the activity benefit; and building and maintaining relationships (https://www.invo.org.uk/wp-content/uploads/2019/04/Copro_Guidance_Feb19.pdf, 26 February 2021).

## 2 METHOD

*Aim*: To reflect on the ways in which COVID‐19‐driven redesign of a codesigned and codelivered priority‐setting exercise informs good practice in coproduction.

*Stakeholders*: The project is a UKRI‐funded partnership of four Leicester communities with several institutional stakeholders: the Leicester chapter of Citizens UK; the University of Leicester (School of Sociology, Public Engagement Team); and the University Hospitals of Leicester (NIHR Leicester Biomedical Research Centre, Leicester Diabetes Centre).

*Ethics*: Arriving at a favourable review of priority‐setting activity from the Medicine and Biological Sciences Research Ethics Committee of the University of Leicester required the project team and University Research and Enterprise Department to engage in extensive negotiation to formalize the accountability and transparency requirements of a project not predicated on the existing governance arrangements and safeguards. This is notable because priority‐setting is generally considered public involvement, not research, and therefore not subject to review, suggesting that the university was risk‐averse concerning coproduction.

The review process considered several ethically sensitive components of the project, from the perspective of the university Research Enterprise and Development department, specifically the following:

1. *The employment of community researchers and appropriate associated governance*: Typically, a researcher needs an academic CV, list of publications and relevant academic qualification. The community researchers’ perceived lack of conventional professional accreditation/qualification, within the strict institutional system of governance, was a particular issue. That we recruited from already engaged groups also posed challenges to HR, who saw this as poor recruitment practice. Initially, there was a requirement for in‐person, on‐site, in office hour employment checks for the community researchers, which could not be waived even if the team paid for an HR team member to attend one of our evening training sessions, but COVID meant that this was conducted on zoom, conveniently forcing an appropriate change in practice.
2. *Facilitation of consent by community researchers*: The role of community researchers in consenting was escalated by the review board to the Research Enterprise and Development team, with whom we negotiated a postal process of consenting. This was administrated by institutional researchers at the prompting of a community researcher, who would notify us of the name and address of a potential participant to whom materials would be posted. The consent form needed to be returned to the institutional researchers before the community researcher could proceed. An interventional study standard ‘Participant Information Sheet and Informed Consent Form’ compliant to ICH GCP standards was required.
3. *Requirement for accountability and training*: Community researchers engaged in pre‐role training and project‐specific team briefing on the consent process.
4. *Process of being interviewed by a community researcher*: A topic guide and evidence of briefing and supervision were required.

Thus, the project challenged existing ethical conceptualizations and the governance arrangements that seek to uphold them as unsuited to the community researcher model. This also highlights the lack of governance arrangement for public involvement and engagement generally, which is not formally subject to any kind of review, by bringing this process under scrutiny usually reserved for research activity.

Written informed consent of interview participants and community researchers was documented.

*Recruitment*: Six trainee representatives were recruited from Leicester communities typically underrepresented in university and hospital research engagement via advertisement through stakeholder engagement projects. The original six representatives, whom we designate ‘community researchers’ to enable distinction from ‘institutional researchers’, reduced to three, due to personal challenges, most of which were related to the pandemic. Community researchers served as our principal route into communities, recruiting participants to the priority‐setting exercise, undertaking interviews and analysing themes in the priority‐setting exercise in collaboration with the institutional researchers.

*Method*: Reflective evaluation was integral to the project bid, producing a total of approximately 8 h of data, comprising the following:

(1) Reflection on project progress and the experience of community researchers as part of three whole‐team meetings, recorded through Zoom, with particular focus on collective learning and emergent models of engagement.
(2) Ten independent reflections to camera by team members, in response to requests for reflections on the training, internal processes (HR, governance, etc.), social distancing and COVID‐safe working, and their general and concluding thoughts, one such provided in writing, all collected between June and October 2020.
(3) Five videoed, facilitated debriefs of community researchers by the project officer collected between late August and early October 2020.
(4) A final team‐based reflective evaluation of the project as a whole hosted by the project officer drawing on previous reflections developed through the course of the project.

Iterative team discussion established consensus on key ‘lessons learned’ findings. As such, the team leveraged the opportunity of establish ‘new ways of knowing’^3^ their experience through discourse, socially constructing the themes that we identify as findings and situating them in the context of wider team knowledge. This allowed us to bring together our diverse skills and experiences to construct our understanding of our experiences.

## 3 FINDINGS

Though technically a finding of the priority‐setting, it is a relevant finding of the project that the participants represented typically underrepresented groups, possibly as a result of a coproduced methodology sensitive to participant need, and working with trusted members of the community as community researchers. The three community researchers (two Asian women, one Black woman), recruited 40 interview participants. Forty per cent of interview participants were women and 20% were men (40% did not reveal their gender). The ethnicity and age of the participants who provided demographic data are shown in the graphs below. A large number of participants did not provide the demographic data, suggesting that the postal consent and baseline system were suboptimal and that an electronic consent and demographic data collection process that prompted a response would have increased quality. This was the project teams’ recommendation to the university, but it was rejected as failing to meet the current ethical requirements for documentation of consent.


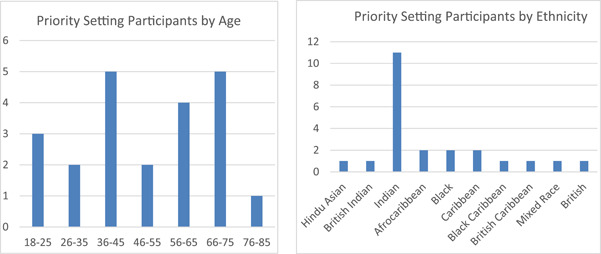


The reflective evaluation enabled us to identify key ‘lessons learned’ to support future coproduction.

We identified *co‐participation as novices and newcomers* as important in establishing the team dynamic. Initial recruitment was successful through projects that already included elements of this, such as participation in dominoes or Bollywood dance classes; importantly, participation as equals or novices helped to dismantle the power dynamics between those of us from an institution and those of us from the community.

The model of engagement involved working with people already involved through engagement via partner organizations’ projects. This, however, proved an efficient way of involving wider community members by establishing the community researchers as nontraditional gatekeepers, and leveraging their connections and social influence to redesign a coproduced project that would work in the pandemic circumstances, and to establish institutional researchers as present within communities. Thus, the institutional components of the team were dependent on the community researchers to engage others within these communities for whom we, within institutions, are hard to reach and who, therefore, seldom hear some community voices. Nonetheless, this approach differs from conventional ‘gatekeeper’ models in drawing from within communities to reach further within those same communities: our community researchers were experts through experience, and not through institutional positionality. This is particularly highlighted by the gender of our community researchers, who are all women, whereas engagement approaches operating through established community and institutional power structures tend to maintain established (male) privilege for example religious organizations.^19^

The effective adaptation of the project demanded the full use of the skills, expertise and strengths of all team members, but particularly highlighted the relevance of the community researchers’ understanding of the real‐world impact of the pandemic on their own communities and ability to therefore predict what data collection methods would, and would not, work in those communities. The community researchers led on the development of the topic guide to maintain the relevance of the project activity to their communities during a time of new and frightening challenges. Institutional researchers then led on the processing of those plans through ethical review and development of an acceptable consenting process. Following discussions, reflections and mutual working deriving from our common experiences as newcomer trainees in the workshops, we iteratively coproduced an approach to ‘citizen research’ that was partly unplanned and had far‐reaching implications.

The unanticipated social and professional changes associated with the pandemic forced the team to foster a reconfiguration of the relationships within the team and between the community researchers, the university and the hospital. Accordingly, an organic example of citizen research emerged not simply as a set of methods, research techniques or practices for successful engagement and collaborative working but also a wide‐reaching and wide‐ranging set of processes and shifts developed via the mutual working through of sincerely foregrounding the principle of treating communities as *constituent partners, not simply ‘target’ participants*.

Where we had originally approached engagement as essentially an ‘inside to out’ process (of ‘reaching out’ to communities), we soon found that it was equally a process of ‘outside to in’ (of renegotiating institutional structures and practices, and partly forcing a review of these). Accordingly, the approach that we developed became far more encompassing than we originally anticipated, forcing a shift, for instance, in the standard operating procedures of how community researchers could be vetted and paid by the university, prompting the development of new ethical safeguards—scalable, multilevel, multistage devolved consent and information processes—and more generally changing the character and form of the intended ‘outputs’ from the study. Whereas the institutional researcher‐designed pre‐COVID plan was heavily focussed on deriving health priorities in different neighbourhoods, the adapted community researcher‐designed plan required us to refocus on exploring how this type of engagement worked and what enabled and obstructed it from success and subsequently to focus, within the university and hospital, on developing systems and processes that at least allowed, and ideally encouraged, coproduction and engagement.

The method of engagement and community research described was not without risks. As previously mentioned, three of the community researchers terminated their involvement in the project. Given the lengthy preimplementation process of training and team building, we were unable to replace them and were unable to capture experiences in two of the Leicester neighbourhoods that we originally sought to explore. With the foundation from the first phase of this study, community researchers from these (and more) Leicester communities could be recruited for a second phase.

## 4 DISCUSSION

The circumstances of the pandemic, as a point of social upheaval to which many institutions reacted by reducing public engagement and involvement, particularly in medical research, and that both reinforced and undermined the authority of medicine and medical research,^20^ created a shift in power dynamics within our project team. The shifting professional and personal responsibilities of team members caused ‘creative disruption’ and inadvertently created the human level of engagement recommended in the NIHR guidance (https://www.invo.org.uk/wp-content/uploads/2019/04/Copro_Guidance_Feb19.pdf, 26 February 2021). The ‘professionals’ were made more human as the demands of their personal lives became apparent, and their professional circumstances shifted suddenly, particularly for personnel who were redeployed to clinical activity or NIHR‐prioritized COVID‐19 research. The impact on community team members varied as some needed to cease commitment to the project to focus on family, and some were now more at liberty to participate, underlining the relevance of social capital identified in the existing literature.^14^, ^18^ The community team members who stayed with the project at this time assumed roles of responsibility and creativity in the project, in a demonstration of passion and compassion seen nationally towards health and science. They rapidly recreated the project to focus on community researcher‐organized remote engagement with other community members, which culminated in them conducting a series of interviews using digital (e.g., video conferencing) and conventional media (e.g., telephone calls) to collate data on health priorities in their communities.

As such, it was the community researchers who initially demonstrated the agility that was fundamental to the enhanced coproductive nature of the project. Community researchers were able to identify key ways of improving research and engagement optimal to their communities during the data collection phase. This was an ongoing process of developing their own practice; for instance, they found that sending questions to interviewees before conducting the interview, allowing time to reflect upon what information they wanted to contribute, was effective.

The project evaluation has, within the limitations of a case study, implications for good practice in coproduction. The findings reflected the requirement of flexibility and experience of inflexibility noted in the literature^1^, ^6^, ^16^, ^18^ and highlighted the need for compatibility of organizational structures with coproduction.^14^, ^21^ Such agility is in some ways alien to health science research, where a strict protocol is followed, and potentially articulates a tension between community and qualitative methods and the more typical quantitative methods of medical research. The role of flexibility is in stark contrast to short‐term funding arrangements that demand rapid implementation. The transformative circumstances of the pandemic dismantled the typical funder requirement of a project plan that, by definition, limits coproduction. This identifies a role for funders in providing not only strong encouragement towards collaboration and coproduction but also guidance on delivery and resourcing.

Barriers to adaptation and function were experienced where there was a lack of agility, generally outside the project team in the proximal infrastructure. The revision of governance arrangements and authorization of community researchers to collect data involved a complex interplay between negotiating institutional processes and safeguards and meeting project objectives, whilst allowing community researchers to play a decisive role in the character and content of the project. The process of adaptation reflected the necessity of adequate management capacity to deliver coproduction,^2^, ^14^ which in turn requires resourcing. A facilitative management style was, for the institutional components of the team, emergent from the process of interaction and coproduction, and fundamental to making things happen. This was also found by Bussu and Bartel in the field of urban planning, and supports the need that they identify for a shift in public service management style towards facilitation.^22^ We needed to think creatively to tackle new and unanticipated challenges in areas outside our expertise in the ‘stovepipes’ of university and hospital administration. This may represent an emergent field of management capacity necessary for coproduction.

Supporting need for resourcing and management capacities creates conceptual implications, challenging the perception of coproduction as a means to reduce public investment.

More widely, the experience reinforced the importance of understanding concepts of power in relationships. Power underpinned the building of horizontal partnerships (as opposed to hierarchical), the potential for open and honest communication (and the most effective ways to achieve this) and the need to foreground community voices in shaping and iteratively reshaping the project.

The project process changed how team members thought about engagement. Our experience highlighted how meaningful engagement necessitates a refiguring of relationships and power between community members, core institutional stakeholders and partner organizations. Our repositioning of community representatives as community researchers (paid, institutionally recognized and affiliated) entailed not just a change of title but also a change in their (and our respective) positionality. We view such reconfiguring relations, in particular, the degree of institutional embeddedness, as not so much the ‘context’ but the ‘stuff’ of engagement, both the medium and the outcome of our pursuit of ‘meaningful and enduring’ engagement in partnership with the community researchers, and the cornerstone of coproduction.

Finally, the project yielded insights into the methodology of priority‐setting. A recognized weakness of priority‐setting, and general public involvement, is that they potentially rate the wants of large or well‐represented groups over the needs of smaller, or seldom‐heard, groups, and thus may exacerbate health inequalities.^23^ The project sought to address this by adapting the methodology of priority‐setting to local needs with our community researchers and stakeholders, and indeed did so on a greater scale than anticipated through coproduced redesign of the project. The sample is clearly skewed towards typically excluded groups, and is a reflection of the project strategy to target underrepresented communities by identifying neighbourhoods from which to recruit.

The project is now in a critical phase. Having developed relationships and a role in communities, it is essential not to suddenly withdraw, potentially exacerbating distrust of health and science at a time where trust in this is essential to an effective response to the global pandemic. The pattern of research and community funding creates a challenge to certainty and confidence to all parties involved and maintains the unequal distribution of power with community researchers who remain in a position of less security in terms of their role.

The project team has identified small pots of funding to support work until August 2021, from other research programmes and from the East Midlands Clinical Research Network Public Engagement small grants. This will be used to conduct further ‘listening projects’ led by the community researchers, notably including an exploration of why people in Leicester think COVID‐19 hit the city so hard. This requires further project adaptation inclusive of the consent processes, methodology and topic guide. The delicate nature of the new discussion topic has provoked re‐enactment of institutional concerns around governance that articulate the persistent institutional resistance to power redistribution. Having established proof of concept as a mode of engagement, we are seeking long‐term financial support to expand the project into a broader range of neighbourhoods, and therefore, more diverse engagement across Leicester.

## CONFLICT OF INTERESTS

The authors declare that there are no conflict of interests.

## AUTHOR CONTRIBUTIONS

The bid for project funding was written by Marie Nugent, Jason Hughes and Rebecca Pritchard. Operational aspects of the project, including supervision of community researchers, were undertaken by Project Officer Dr. Furaha Asani. Community researchers were trained and in some cases recruited by Leicester Citizens personnel Raheema Caratella, Keith Hebden and Daniel Mackintosh. Prafulla Modi, Sonal Bhavsar and Pamela Campbell‐Morris designed and undertook the COVID‐adapted data collection, and reported back themes to FA, who collated data. All authors were involved in drawing conclusions, the evaluation of the project and the writing of this article.

## Data Availability

The raw data for the evaluation can be accessed by researchers with appropriate ethical and institutional approval on approach to the lead author.
